# 98179 Identifying Low-Value Care Across A Statewide Health System: Collaboration Between Quality, Population Health, Informatics, and Health Services Research

**DOI:** 10.1017/cts.2021.720

**Published:** 2021-03-30

**Authors:** Carlos Irwin A. Oronce, John N. Mafi, Ray Pablo, Andrea Sorensen, Ayan Patel, Lisa Dahm, Samuel A. Skootsky, Rachael Sak, Catherine Sarkisian

**Affiliations:** 1 Greater Los Angeles VA Healthcare System and UCLA David Geffen School of Medicine; 2 UCLA David Geffen School of Medicine; 3 University of California Health; 4 UCLA Health

## Abstract

ABSTRACT IMPACT: This project demonstrates that addressing low-value care, which has the potential to cause patient harm, relies on novel data tools and collaboration between health system and research stakeholders. OBJECTIVES/GOALS: Reducing low-value care, or patient care that offers no net benefit in specific clinical scenarios, is an important approach to improving value as it can simultaneously lower health care spending and improve quality. We describe an initiative to identify such care in a large statewide employer. METHODS/STUDY POPULATION: Claims data for self-funded University of California (UC) Preferred Provider Organization (PPO) plan members during 2019 were abstracted from the University of California Health (UCH) Clinical Data Warehouse, a unique central database that includes electronic medical record data from >5 million patients across UC medical campuses and all claims from UC self-funded health plans. UCH spans six academic health systems across California. The Milliman MedInsight Health Waste Calculator, a proprietary algorithm-based software tool, was used to identify low-value care and estimate associated spending. The HWC measures 48 low-value services using recommendations from the Choosing Wisely Campaign, the US Preventive Services Task Force, and other clinical specialty guidelines. RESULTS/ANTICIPATED RESULTS: Of 43,882 members of the UC PPO, 11,174 (25.4%) received at least one low-value service. The HWC identified 50,103 eligible services and classified 35% as low-value. Total spending on low-value services ranged between $2,209,516 and $5,089,866, based on a more or less conservative estimate. Across the five sites, the proportion of low-value services ranged from 31% to 39%. Five services comprised 65% of costs from low-value care: annual EKGs, preoperative baseline labs for low-risk surgeries, vitamin D deficiency screening, imaging for eye disease, and headache imaging. The top five services by order frequency were annual EKGs, vitamin D tests, preoperative labs, antibiotics for upper respiratory infections, and imaging for eye disease. DISCUSSION/SIGNIFICANCE OF FINDINGS: Low-value care is prevalent and costly within a large statewide employer. Collaborative multidisciplinary partnerships between employers, health systems, informatics, and researchers can leverage existing data to identify opportunities for improving the value of care for covered populations.

